# Toxins VapC and PasB from Prokaryotic TA Modules Remain Active in Mammalian Cancer Cells

**DOI:** 10.3390/toxins6102948

**Published:** 2014-09-30

**Authors:** Łukasz Wieteska, Aleksander Skulimowski, Magdalena Cybula, Janusz Szemraj

**Affiliations:** Department of Medical Biochemistry, Medical University of Lodz, ul. Mazowiecka 6/8, 92-215 Lodz, Poland; E-Mails: alexander1@op.pl (A.S.); magdalenaa.cybula@gmail.com (M.C.); jszemraj@csk.umed.lodz.pl (J.S.)

**Keywords:** toxin-antitoxin, cancer cells, immunotoxin, VapC, PasB

## Abstract

Among the great number of addictive modules which have been discovered, only a few have been characterized. However, research concerning the adoption of toxins from these systems shows their great potential as a tool for molecular biology and medicine. In our study, we tested two different toxins derived from class II addictive modules, *pasAB* from plasmid pTF-FC2 (*Thiobacillus ferrooxidans*) and vapBC 2829Rv (*Mycobacterium tuberculosis*), in terms of their usefulness as growth inhibitors of human cancer cell lines, namely KYSE 30, MCF-7 and HCT 116. Transfection of the *pasB* and *vapC* genes into the cells was conducted with the use of two different expression systems. Cellular effects, such as apoptosis, necrosis and changes in the cell cycle, were tested by applying flow cytometry with immunofluorescence staining. Our findings demonstrated that toxins VapC and PasB demonstrate proapoptotic activity in the human cancer cells, regardless of the expression system used. As for the toxin PasB, observed changes were more subtle than for the VapC. The level of expression for both the genes was monitored by QPCR and did not reveal statistically significant differences within the same cell line.

## 1. Introduction

Toxin-antitoxin systems are widely distributed among prokaryotes. They are often present even in multiple copies in the bacterial chromosome [1]. Toxin-antitoxin systems can be divided into five classes. The first one includes systems that are composed of protein toxin and complementary to the toxin’s mRNA non-coding RNA, which constitutes antitoxin. The translation of toxin mRNA is inhibited by duplex formation. The majority of TA belongs to the second class, which is formed by systems composed of toxins and antitoxins that are small proteins (90–130 amino acids)-the toxin is stable whereas the antitoxin is labile. The antitoxin has a strong affinity to the toxin and, in connection with it, causes the complete loss of toxin activity [2]. The third class includes systems in which the toxic protein is directly bound and inactivated by non-coding RNA antitoxin [3]. New classes IV and V contain so far only one representative. YeeU-CbtA is the only system in which antitoxin (YeeU) does not interact directly with the toxin (CbtA), but stabilizes the toxin target protein and prevents binding of CbtA to MreB and FtsZ proteins, which are enrolled in maintaining cellular shape and cell division [4,5]. In addition, distinct properties of GhoT-GhoS system determined constitution of new fifth class. In this case, stable antitoxin GhoS has endoribonucleatic activity and specifically cleaves mRNA of the Toxin GhoT, preventing its translation [6]. Toxin–antitoxin systems can be found in plasmids, where they act as the plasmid maintaining system, or in genomic DNA, where they are involved in the prevention of genome-wide scale deletions [7], response to stressful conditions, biofilm formation, or as discussed recently, programmed cell death [8,9]. The majority of toxins act as specific RNAs; however, there are also systems in which toxin interferes with DNA replication, peptidoglycan synthesis, cell division or disrupts inner membrane [7,8]. It should be mentioned that mechanism of action of many systems predicted by bioinformaticians still remains unknown [1,9,10].

There are only a few studies that report activity preservation of toxins derived from TA modules in mammalian cells. Audoly *et al.* [11], demonstrated that toxin VapC originated from *Rickettsia felis* is active in L929 murine cells and proved its role in rickettsial infection. It is believed that multiple loci of *vapBC* in the rickettsial genome are responsible for host cell apoptosis [11]. A similar finding, stating that the presence of the *vapBC* system increases virulence, seems to be true for *Haemophilus influenza* as well [12]. Results published by Yamamoto *et al.* [13], showed that the overexpression of RelE toxin from the other well described system, RelBE from the chromosome of *E. coli* K12 in A-549 lung cancer and TREx-U2OS osteosarcoma cells, can lead to death through the apoptosis pathway. In addition, de la Cueva-Mendez and colleagues demonstrated that toxin Kid from parD system originated from plasmid R1 when injected into HeLa and SW480 cells dramatically decrease their survival [14]. One of the most studied and promising system has been mazEF derived from *E. coli* chromosomal DNA [15,16,17,18,19]. Current research on mazEF has found its great potential in developing new strategies for antiviral therapies [20]. The MazF toxin was used to construct a recombinant drug against HCV. The MazF toxin was fused to inhibitor, which could be degraded in contact with the NS3 protease encoded by the HCV RNA. As a result of the activated toxin action, infected cells died preventing the spread of the virus [21]. Work is also underway on therapy against HIV. Okamoto *et al.*, managed to significantly reduce the level of viral transcripts without damaging T lymphocytes [22]. The above cases suggest that toxins derived from toxin-antitoxin modules can be of great importance to medicine and medical science. Thus, it is important to learn more about their functions, possible interactions and implementations. In our study, we focused on two toxins which belongs to the II class of TA: VapC 2829 derived from the chromosome of *Mycobacterium tuberculosis* H37Rv and PasB originated from plasmid pTF-FC2 from *Thiobacillus ferroxidans*, and their proapoptotic activity in diverse human cancer cell lines.

## 2. Results and Discussion

### 2.1. Properties of Addictive Modules Chosen for Study: PasAB and VapBC

**Figure 1:**
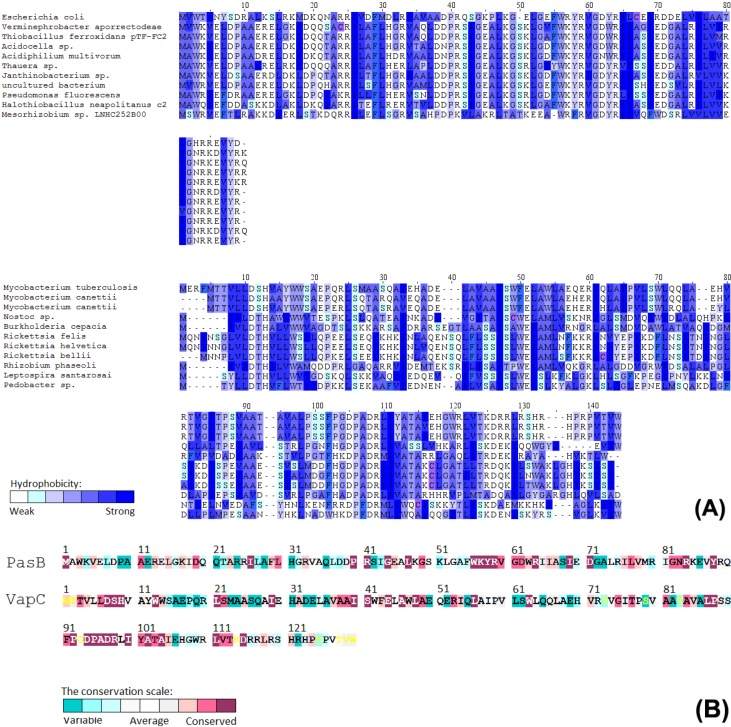
(**A**) Multiple sequence alignment of VapC and PasB with most similar homologues and described family members. PasB (upper MSA) shows standard homology within RelE family (46% identity to the well-studied toxin from *Escherichia coli*). However, there are visible regions within active site that are unique and conserved among PasB like sequences. Strong homology can be observed for other non-related families, suggesting horizontal gene transfer. Color gradient corresponds to different amino acid hydrophobicity; (**B**) Conservation pattern calculated for protein families (for over 300 representatives).

Toxin-antitoxin system *PasAB* located on plasmid pTF-FC2, originated from *Thiobacillus ferroxidans* [23]. It belongs to the type II TA systems, in which both partners, toxin and neutralizing antitoxin, are proteins [1]. The system is said to belong to the RelBE family [24]. However, the psi-blast analysis shows that the sequence similarity of PasAB to other RelBE family shows significant changes within active site (Figure 1). Nonetheless, it remains fully functional when transformed to *E. coli* [25]. Toxin-antitoxin *vapBC* systems belong to type II TA and are among the most abundant systems which encode the PIN domain (PilT *N*-terminus domain), associated with the nuclease activity [25]. *Mycobacterium tuberculosis*, among many others types of TA modules, contains over 45 *vapBC* loci [26]. It was shown for *vapBC* systems derived from *Shigella flexneri* virulence plasmid pMYSH6000 and *Salmonella enterica serovar Typhimurium LT2* that they act as specific tRNAses [27]. Like other type II TAs, antitoxin *VapB* inhibits cognate toxin by direct protein-protein interaction. For our research, we chose the *vapBC* 2829Rv-2830Rv system derived from *Mycobacterium tuberculosis* H37Rv, which was previously tested to be one of the most potent growth inhibitors when expressed in *Mycobacterium smegmatis* [2]. The psi-blast analysis showed that genes with complete sequence identity are widely distributed among *Mycobacterium tuberculosis*, *Mycobacterium bovis* and *Mycobacterium africanus* strains (Figure 1).

### 2.2. Effect of Induced Toxin Expression in Human Cancer Cell Lines

**Figure 2:**
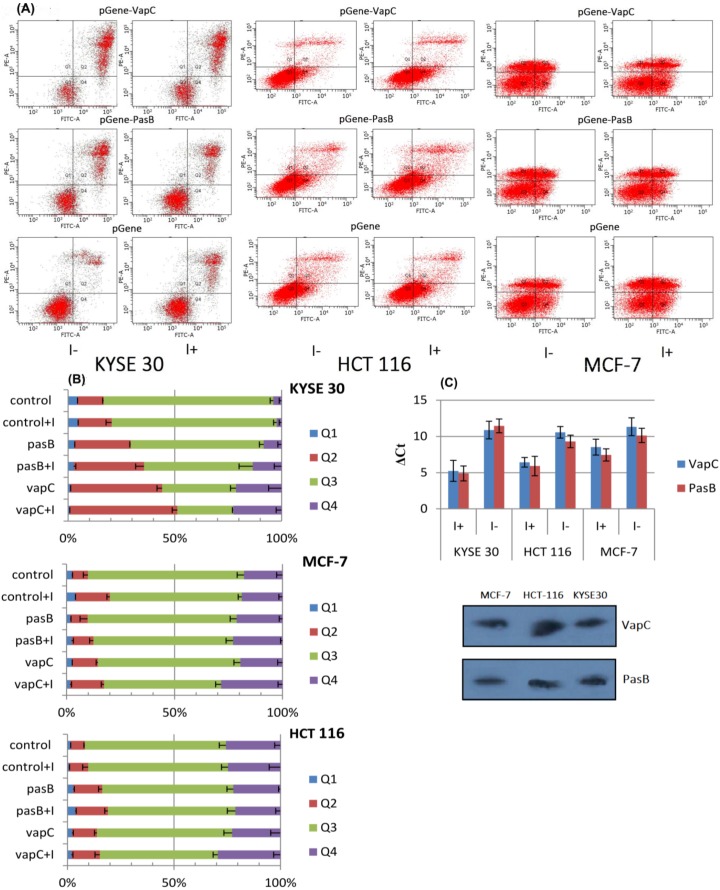
(**A**) Cytometry analysis of transfected cells. Visible differences in population Q1–Q4, depending on the construct used for transfection (*pGene* -control, *pGene*-PasB, *pGene*-VapC) and addition of mifepristone (I+). Q1—necrotic cells, Q2—late-apoptotic cells, Q3—cells alive, Q4—early apoptotic cells. Average of two experiments performed in triplicate; Quantitative data is presented on the histogram in section (**B**); Histogram (**C**) shows differences in the level of mRNA for toxins VapC and PasB with (I+) and without (I−) mifepristone; Bottom: western blot showing bands of expressed toxins (after induction) in all tested cell lines.

In our research, we checked the influence of the endogenous expression of toxins PasB and VapC on apoptosis and necrosis induction in various cell lines: MCF-7 human breast cancer, KYSE30 human squamous cell carcinoma and HCT-116 human colon cancer. For induced expression, the GeneSwitch system (Invitrogen, Carlsbad, CA, USA) was selected (see Experimental Section). Clones positively expressing regulatory protein GAL4-DBD/hPR-LBD/p65-AD were transfected with plasmid *pGene*, *pGene*-PasB and *pGene*-VapC. After transfection, the expression of inserted genes was induced by adding mifepristone. Cell conditions were tested with flow cytometry (see Section 3.7) 72 h after induction (Figure 2). Due to clear and repeatable differences observed between the numbers of apoptotic, necrotic and live cells transfected with the control vector between different cell lines, acquired data had to be analyzed separately. As for an increase in the number of apoptotic cells compared to the control vector in the KYSE30 line, we noticed a statistically significant change for cells transfected with the *vapC* toxin gene. The difference was detectable even without protein induction (*p* < 0.0001), but correlated more accurately after induction. Comparison between populations of late and early apoptotic cells revealed significantly higher numbers in the early apoptotic population. Nevertheless, the overall proportional change was higher for cells being in the late apoptosis phase. That observation was applicable to both genes. When comparing controls of HCT-116 to KYSE30 cells, we could observe a higher number of early apoptotic cells. That fact can be explained by higher sensitivity to the transfection agent. As for the comparison between the numbers of apoptotic, necrotic and viable cells for the HCT-116 line, there was a statistically significant difference between cells transfected with *pGene*-VapC and controls (*p* < 0.009). That change occurred mostly for the late apoptotic population. For cells transfected with *pGene*-PasB, we could also observe a statistically significant change in the number of late apoptotic cells; the dependence, however, was much weaker (*p <* 0.022). That effect was not remarkably increased by induction. MCF-7 cells responded in a similar way to the HCT-116 line. Only cells transfected with *pGene*-VapC exhibited a statistically significant increase in the number of late apoptotic cells and drop for the viable population (*p <* 0.003 and *p <* 0.017 respectively). In MCF-7 cells, induction with mifepristone caused most remarkable differences comparing to control. It is known that MCF-7 cells express receptors for glucocorticoids, which can interfere with mifepristone [28]. In our stable transfected cells expressing regulatory protein for mifepristone regulation, however, we could still distinguish between induced cells transfected with the control vector and the vector with the cloned *vapC* gene.

To test the hypothesis that observed effects are not disrupted by different level of protein expression, we measured the mRNA level for each cloned *pasB* and *vapC* gene for induced and not induced cells. We were able to detect mRNA in both cases. There was, however, an over 10-fold change in expression after mifepristone induction (mean Δ*Ct* 4.17 ± 1.12). Additionally, we performed qualitatively western-blot analysis (Figure 2). In each case, we could detect band representing expressed fusion protein.

### 2.3. Toxin Expression with the EGFP Tracking System

**Figure 3:**
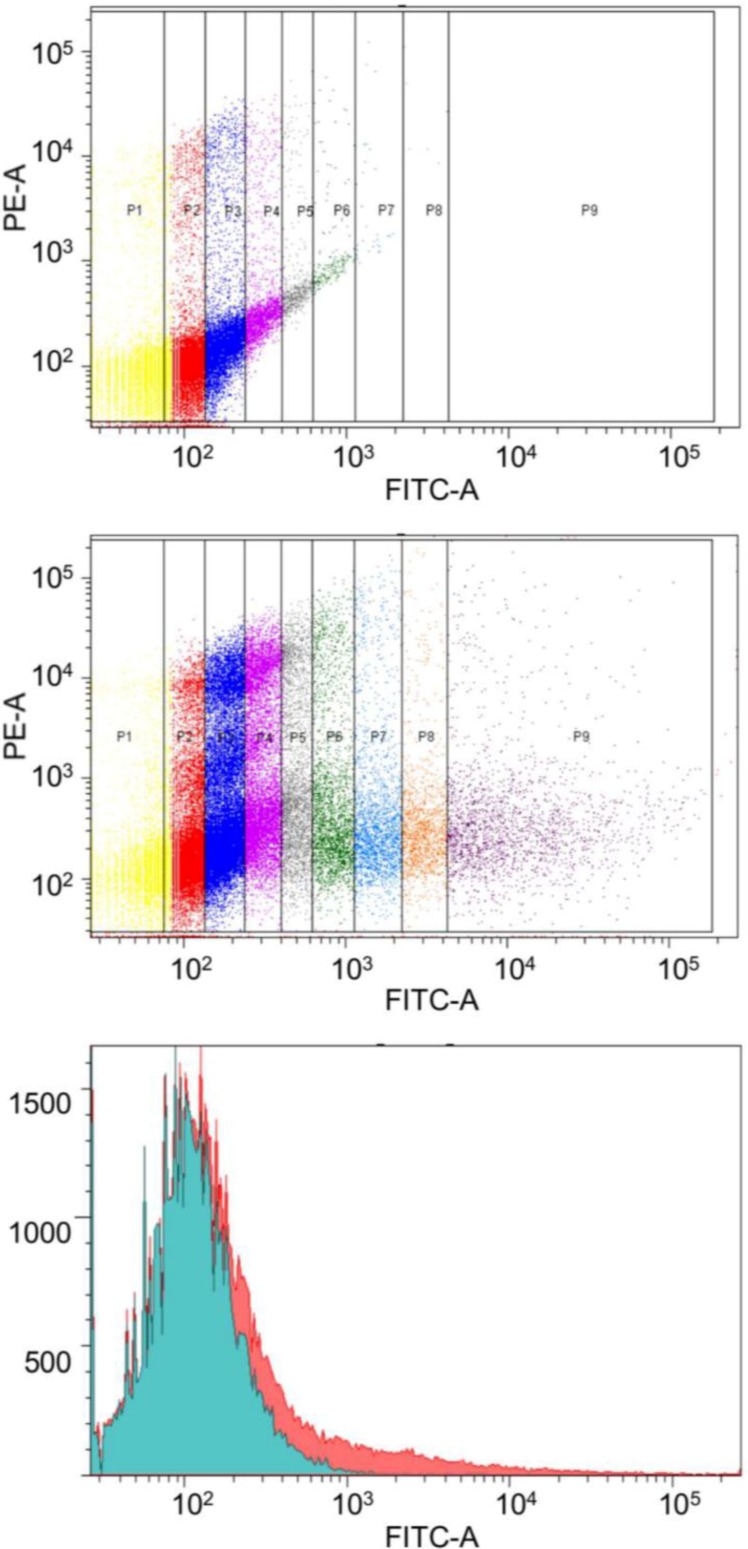
Procedure for the flow cytometry analysis with EGFP marker gene. The top two graphs show division into compartments depending on signal strength for the channel FITC-A (signal from EGFP). The apparent difference between the cells transfected with the vector containing the EGFP protein (**upper**) from the control vector (the **middle** graph). Compartments from P2 to P8 are of equal width. P1 and P2 are located on the borders, their width was dependent on the maximum (minimum) capacities event registration by the instrument. **Lower** chart shows the quantitative difference between the number of cells in particular the FITC channel of transfected to untransfected cells.

**Figure 4:**
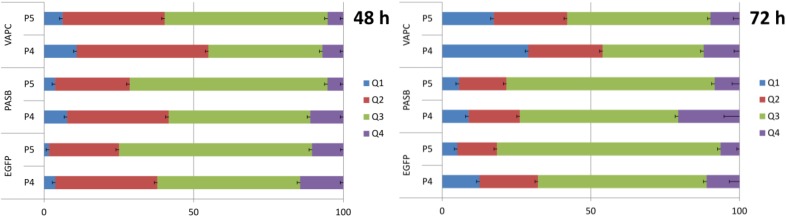
Distribution of cells transfected with constructs pEGFP (control), pEGFP-vapB and pEGFP-VapC among populations Q1–Q4 (after 48 h and 72 h since transfection). Results shown for compartments P4 and P5, where changes were most significant.

For brief confirmation, we tried to reproduce similar effects using a different expression system. The vector pBudCE4.1 which contains the human cytomegalovirus (CMV) immediate-early promoter and the human elongation factor 1α-subunit (EF-1α) promoter for the independent expression of two recombinant proteins was selected. Constructs containing simultaneously one of the tested toxin genes (*pasB*, *vapC*, cloned under EF-1α promoter) and the gene of Enhanced Green Fluorescent Protein (EGFP) were constructed, which enabled us to measure transfection rate more accurately. KYSE 30 and MCF-7 cells stained with Annexin-V-Pacific blue and PI were tested with flow cytometry 48 h and 72 h after transfection. During data analysis, we grouped cells into 9 clusters according to the level of EGFP signal (measuring signal strength in the FITC-A channel) Apoptotic, necrotic and viable cell populations were calculated separately for each cluster (Figure 3). Figure 4 present results for clusters P4 and P5 in which changes in population ratios were the most remarkable. For cells transfected with pEGFP-VapC 48 h after transfection, there was a clearly visible drop in the number of viable cells compared to control (*p <* 0.005) and simultaneous increase in the late apoptotic cell population. This propensity was also visible 72 h after transfection with an additional increase in the necrotic population (*p <* 0.005). There was no visible difference between control and cells transfected with pEGFP-PasB. A similar observation was made for the MCF-7 cell line (data not shown), but with lower statistical significance (*p <* 0.04).

### 2.4. Cell Cycle Analysis

**Figure 5:**
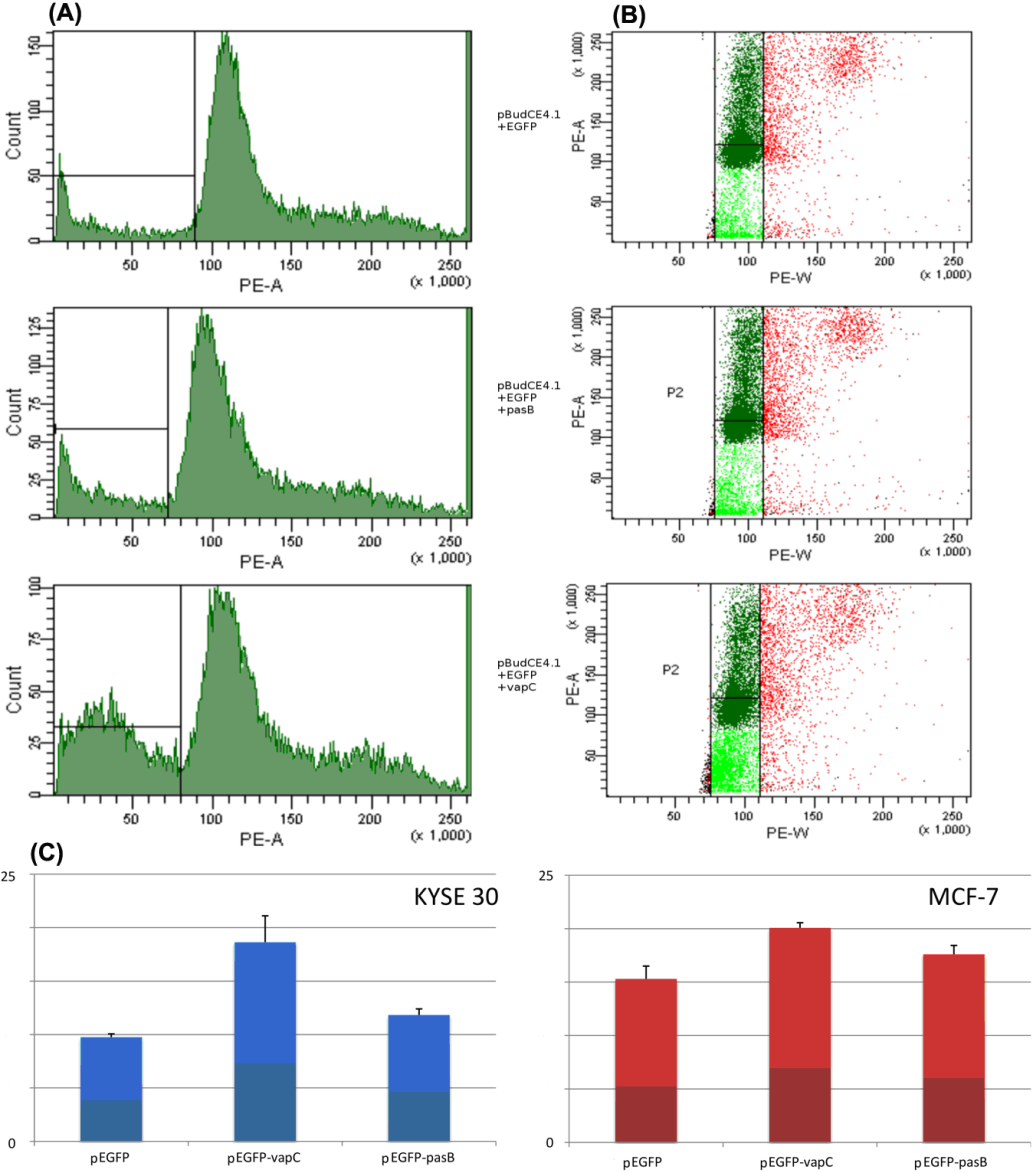
Cell cycle analysis of cells transfected with constructs: pEGFP-PasB, pEGFP-VapC and control *pEGFP*. Histograms show propidium iodide signal strength (**A**) and population range (**B**) taken into analysis. Visible increase of Sub-G1 population, mostly for cells transfected with pEGFP-VapC. (**C**) histograms showing number (%) of cells in Sub-G1 phase (visible as light green on plots in section (**C**)).

Due to the fact that both PasB and VapC act as specific RNAses, their expression should not have affect the cell cycle. In addition, the sub-G1 population responsible for apoptotic cells was expected to be expanded. To confirm that hypothesis, we analyzed the cell cycle of KYSE 30 and MCF-7 cell lines 72 h after transfection with pEGFP-PasB, pEGFP-VapC and pEGFP as the control vector (Figure 5). After transfection with pEGFP-VapC, the sub G1 population increased remarkably for KYSE 30 and MCF-7 cells (*p <* 0.002 and *p <* 0.0001 respectively). There was also a statistically significant increase in the sub-G1 population for MCF-7 cells transfected with pEGFP-PasB (*p <* 0.02). That effect, however, was approximately two times weaker compared to pEGFP-VapC and not detectable in the KYSE 30 cell line. Among the tested lines, KYSE 30 appeared to be the most susceptible to VapC expression. That line behaved most stably and the reproducible results were easier to obtain. In similarly designed research, susceptibility variations among cell lines were also observed [28]. As for the apoptosis induction potency of the PasB from plasmid pTF-FC2, according to our results, two out of three experiments performed produced a statistically significant drop in viable cells and apoptosis induction, but induced changes were more subtle than those observed for VapC. Importantly, changes in the potency were not caused by the different levels of expression which were monitored with the QPCR method and did not reveal significant disparity. The RelE toxin cleaves ribosomal A-site of the mRNA in prokaryotes and eukaryotes and the mRNA cleavage pattern is conserved within family; however, the sequence alignment with PasB shows major changes within the active site of the toxin; thus, more research into PasB biology is needed. It is worth noticing that a toxin whose activity depends on the interaction not only with the mRNA but also with the ribosome, remains active in eukaryotic cells. This effect was also observed for the RelE toxin from *Escherichia coli* and it was demonstrated that it occurs by the identical mechanism of action [29]. Authors claim that this observation supports the view that the structural organization of the A site of the small ribosomal subunit is very similar in bacteria and mammals [29].

The use of the GeneSwitch system (Invitrogen, Carlsbad, CA, USA) allowed us to trigger the expression of transfected genes. Mifepristone induction, however, did not appear to be neutral to the cell viability, As viability also varied between particular clones stably expressing GAL4-DBD/hPR-LBD/p65-AD, it was thus difficult to establish comparison between straight lines. In addition, even when the mifepristone was absent, every clone tested exhibited a clearly detectable level of basal expression. The use of the pBudCE.4.1 system (Invitrogen, Carlsbad, CA, USA) enabled us to confirm our previous observations. Dividing cell populations by EGFP signal strength greatly improved the sensitivity of cytometric assay, but changes were visible even when calculated collectively. It could appear that the population which exhibited the highest signal of EGFP should be most affected by the simultaneous toxin expression. An explanation for that phenomenon might be the fact that cells affected by toxic RNAses diminish the level of total expression.

## 3. Experimental Section

### 3.1. Gene and Cell Line Sources

Plasmid pTF-FC2, kindly provided by D.E. Rawlings, was the source of the *pasAB* system, while genomic DNA of *Mycobacterium tuberculosis* H37Rv, kindly provided by J. Dziadek, was the source of the *vapBC* 2829-2830 system. The gene of EGFP was amplified from plasmid pEGFP-n1 from the local vector library. The HCT-116 line expressing regulatory protein GAL4-DBD/hPR-LBD/p65-AD was kindly provided by Fu-Ming Tsai [30]. MCF-7 and KYSE 30 lines were previously purchased from ATCC (ATCC, Manassas, VA, USA).

### 3.2. Construct Preparation

In this study, two different eukaryotic expression vectors were used: pBudCE4.1 (Invitrogen, Carlsbad, CA, USA) allowing for the simultaneous expression of two different genes and the GeneSwitch System (Invitrogen, Carlsbad, CA, USA) enabling induced expression in mammalian cells. For each gene in each system, two or, when needed, three different constructs were prepared: (I) native kozak sequence + gene + stop codon; (II) native kozak sequence + gene + myc/V5 epitope; (III) enhanced kozak sequence + gene + stop codon. All genes were amplified from templates with HiFi high fidelity polymerase (Kappa Biosystems, Wilmington, MA, USA) and cloned into Multi Cloning Site (MCS) of chosen vectors with the use of FastDigest restriction enzymes (Thermo Scientific, Vilnius, Lithuania). Ligation reactions were performed with the Rapid Ligation and Dephos system (Roche, Mannheim, Germany). All clones were prepared in XL10 *E. coli* chemically competent cells using the standard protocol [31]. All media plates were supplemented with proper antibiotics (zeocin at 50 mg/mL, ampicillin 100 mg/mL).

### 3.3. Cell Cultures

HCT-116 (human colon cancer) was maintained in McCoy’s 5A (Sigma-Aldrich, St. Louis, MO, USA) supplemented with 10% fetal bovine serum (FBS). MCF-7 (human breast cancer) was maintained in Eagle’s Minimum Essential Medium (EMEM) (Sigma-Aldrich, St. Louis, MO, USA) supplemented with 20% FBS, 1% non-essential amino acids and 200 mM l-Glutamine. KYSE30 (human squamous cell carcinoma) was cultured in RPMI 1640, HAM’s F12 (1:1) mediums (Sigma-Aldrich, St. Louis, MO, USA) supplemented with 2% FBS and 2 mM l-Glutamine. Cell cultures were maintained at 37 °C under 5% CO_2_ in an incubator.

### 3.4. Stable Transfection with Regulatory Vector pSwitch

Ninety percent confluent cells cultured in a 25 cm^2^ flask were transfected with 10 μg of linearized vector pSwitch (Bst1107 I restriction endonuclease) using 25 μL of lipofectamine 2000 (Invitrogen, Carlsbad, CA, USA). After 24 h cells were trypsinized (Sigma-Aldrich, St. Louis, MO, USA), harvested by centrifugation (2000 rpm, 4 min) and plated at 5 × 10^5^/well on a 6 well plate. Cells were allowed to adhere overnight and a fresh medium supplemented with appropriate concentration of hygromycin was applied. The optimal concentration of hygromycin was determined according to the manufacturer’s protocol. The medium was replaced every 3 days. After three to four weeks, separate colonies could be visible. Cells from different colonies were moved to new 96 well plates and tested by transfection with specially constructed vector *pGene*-EGFP and mifepristone induction. Clones that showed the same morphology as the parent strain, minimal production of EGFP without induction and high EGFP level after induction with mifepristone were assigned for further investigation.

### 3.5. Transfection with the pGene Vector and Induction

Cells were plated on 24 well plates (50,000 cells/well) and left overnight to adhere. Next day the medium was replaced and plasmid DNA/lipofectamine complexes were added to each well (0.5 μg of plasmid DNA, 2 μL of lipofectamine 2000/well). After 24 h the medium was replaced and mifepristone was added to the final concentration of 10 nM. Flow cytometry analyses were performed 48 h and 72 h after induction.

### 3.6. Transfection with the pBud4.1CE System

Cells were plated on 24 well plates (50,000 cells/well) and left overnight to adhere. Next day the medium was replaced and plasmid DNA/lipofectamine complexes were added to each well (0.5 μg of plasmid DNA, 2 μL of lipofectamine 2000/well). After 24 h the medium was replaced. Flow cytometry analyses were performed 48 h and 72 h after induction. During the transfection procedure no antibiotics were used. All experiments were done in triplicates and two repeats.

### 3.7. Apoptosis, Necrosis and Viable Cell Assay

Cells after transfection (72 h and 96 h) were trypsinized, harvested, washed twice with PBS and resuspended in binding buffer at the concentration of 10^6^ cells/mL. 100 μL were transferred to a new tube and 5 μL of Annexin-V FITC (for the GeneSwitch system) or Annexin-V Pacific blue (for pBuD4.1CE with EGFP marker protein) with 5 μL of propidium iodide were added. After incubation in the dark for 15 min, the cells were subjected to flow cytometry analysis with Canto II (BD Bioscience, San Diego, CA, USA). All necessary controls were also included (untransfected cells, cells stained only with PI or Annexin-V). Unstained cells were consider as viable. Cells stained only with propidium iodide were considered to be necrotic. Cells stained only with Annexin-V FITC or Annexin-V Pacific blue were considered as early apoptotic. Cell stained both with propidium iodide and Annexin-V FITC or Pacific blue were considered as late apoptotic.

### 3.8. Cell Cycle Assay

Cells after transfection (48 h, 72 h and 96 h) were trypsinized, harvested, washed twice with PBS, resuspended in ice cold 70% ethanol at the concentration of 10^6^ cells/mL and stored in −20 °C for at least 24 h. On the day of analysis, cells were washed twice with PBS and resuspended in PBS supplemented with 0.5 mg/mL RNAse and 50 μg/mL propidium iodide. After 2 h of incubation the cells were analyzed with flow cytometry at a low flow.

### 3.9. Gene Expression Assays

Cells were cultured in 25 cm^2^ at 1,000,000/flask. When the confluence reached 90%, the cells were transfected with proper constructs in *pGene* or pBud4.1CE systems according to the standard protocol (10 μg plasmid DNA, 25 μL of lipofectamine 2000). After 72 h the cells were harvested and total RNA was isolated according to the standard TRI reagent protocol with an additional DNAse digestion step. cDNA synthesis was performed using the SuperScript® III First-Strand Synthesis System (Invitrogen, Carlsbad, CA, USA) and QPCR was performed with specially designed primers. As a reference, we used 66 nucleotide sequence from GAPDH gene amplified with following primers: Forward 5'AGCCACATCGCTCAGACA3'; reversed: 5'GCCCAATACGACCAAATCC3'. For western blotting, cells were cultured and transfected as described above. After 72 h the cells were harvested and resuspended on ice in lysis buffer (20 mM Tris-HCl pH 7.5, 150 mM NaCl, 1 mM EDTA, 1% Triton) supplemented with the Protease Inhibitor Cocktail (Promega, Madison, WI, USA). Cells were frozen and thawed repeatedly three times and centrifuged for 30 min at 15,000 rpm at 4 °C. Supernatant was collected and 12.5% SDS-PAGE gel was run. Membrane transfer and monoclonal antibody binding to His and V5 tag (Serotec, Kidlington, UK) procedures were done according to the standard protocol [30]. The blots were developed with Immun-Star HRP Chemiluminescent Kit (Bio-Rad, Hercules, CA, USA) and registered on KODAK BioMax MS film (Kodak, Rochester, NY, USA).

## 4. Conclusions

Recent work on *vapBC*, *mazEF*, *relBE* and other toxin-antitoxin systems has provided more information about their mechanisms of action and possible application in eukaryotic machinery [14,16,21]. Moreover, the usage of the toxin from TA in gene therapy seems to be very promising. More research in this field is, however, still required. In our study, we were able to prove for the first time that the VapC toxin derived from the chromosome of *Mycobacterium tuberculosis* preserves its activity in human cancer cell lines KYSE 30 (oesophagus cancer), HCT-116 (colon cancer) and MCF-7 (non-invasive breast cancer), causing statistically significant apoptosis induction.
